# Effects of black tea consumption and caffeine intake on depression risk in black tea consumers

**DOI:** 10.4314/ahs.v21i2.47

**Published:** 2021-06

**Authors:** Esma Asil, Mustafa Volkan Yılmaz, Hulya Yardimci

**Affiliations:** Ankara University, Nutrition and Dietetics

**Keywords:** Caffeine, black tea, beck depression inventory, depression

## Abstract

**Background:**

The aim of this study was to compare black tea consumption and caffeine intake with depression status.

**Subjects and Methods:**

This study was conducted on 491 adults (M:169, F:322). The average daily caffeine intake of individuals was calculated using the amounts of caffeinated beverages they consumed daily and the caffeine contents of these beverages. The participants' depression status was determined using the Beck Depression Inventory (BDI). All of the research data were evaluated using STATA.

**Results:**

According to BDI scores, 30.1% of participants had depression. Black tea was consumed by all of the participants and also had the highest consumption level of 620.1±90.4mL and the mean caffeine intake of the participants was 629.5±418.8 mg. Multivariate regression analyses showed that consuming more than 1 cup was protective against depression up to 4 cups. Moreover, a 450–600 mg caffeine intake also reduces the risk of depression than lower or higher intake levels.

**Conclusion:**

Our study suggests that black tea consumption up to 4 cups and caffeine intake between 450–600 mg can help protect against depression. Further studies are needed to better understand the protective effects of black tea and caffeine on depression.

## Introduction

Caffeine (1,3,7-trimethylxanthine) is a stimulator bioactive component of beverages such as coffee, tea, and energy drinks, as well as some foods like chocolate 1. Caffeine is among the most widely ingested psychoactive substances 2 and studies have found that it may contribute to psychiatric problems including depression 3,4. Depression is a common mental disorder that, by definition, is symptomized by a deepening of one's mood, a loss of interest or enjoyment in life, a decrease of vigor, feelings of guilt or low self-esteem, a lack of appetite, an inability to achieve and maintain natural sleep, and a reduction in the ability to concentrate. Thus, depressive symptoms and disorders are prevalent in populations around the world. The World Health Organization (WHO) states that between 2005 and 2015 there was an increase of 15% in cases of depression and that more than 300 million people (4.4% of the world's population) suffer from depression^5^. Consistent with global data, the WHO indicates that the prevalence of depressive disorder among the population of Turkey, unstandardized for age, is 4.4% 5. The prevalence of depression in adults were between 14.7%–25.8% in various studies conducted in Turkey 6–8.

There are multiple studies evaluating the relationship between caffeine and depression in different populations^4^,^9^–^12^. Lucas, et al.^4^ reported that depression risk decreased with the increase of caffeinated coffee consumption among women in the U.S. In a meta-analysis of studies about the relationship between coffee consumption and depression, consumption of up to 600 mL of coffee demonstrated an association with the reduced risk of depression 14. Guo, et al.^15^ found that individuals who consume four or more cups of coffee per day had a significantly lower risk of depression than those who consume one cup of coffee or less. Similarly, a study conducted in Finland with 2011 participants found that individuals who consumed tea on a daily basis showed fewer symptoms of depression^13^. It was determined that the high consumption of green tea within Japanese society was correlated with a low presence of depressive symptoms 10. In a recent study, Yao, et al.,^16^ using the data from Chinese Longitudinal Healthy Longevity Survey, found that a 1 cup or more level of tea consumption may protect against depression in the elderly.

Black tea consumption is prevalent in Turkey 17,18. While in the early 2000s Turkey ranked third (2.3 kg per capita) after Northern Ireland and England in black tea consumption 19 today, Turkey is leading the world with consumption of approximately 3.2kg per capita 20. Studies show black tea is the main source of caffeine consumption in Turkey 17,18,21.

Understanding the impact of beverages that contain high levels of caffeine, which are widespread and consumed in relatively large amounts, on depression experienced by individuals is important for public health. However, research in Turkey on the relationship between depression and the consumption of caffeinated beverages to date is insufficient. The objective of this study is to expand this knowledge by comparing the consumption levels of black tea and the levels of caffeine intake with the depression status in adults.

## Method

This study was conducted between December 2014 and February 2015 with the participation of 491 office workers (males: 169, females: 322; 33.5±10.9 years) living in Ankara. Black tea drinkers who consumed at least 100 mL of black tea per day were included in the study. Subjects who had a history of anxiety or depression and those who were using antidepressants were excluded from the study. The Beck Depression Inventory (BDI) was used to determine depression. Approval for the research was acquired from the Ethics Board of Ankara University (27/11/2014- 179/1345). Each participant in the research signed a consent form prior to his or her inclusion in the study.

Participants filled out a questionnaire that included socio-demographic (eg. education, income), anthropometric (eg. weight, height), and medical (eg. last measured systolic, diastolic blood pressure) information. The body weights and heights of the individuals who participated in the study were measured in accordance with the technique 22. The body weights of the participants were measured with the Arzum AR580 device, while their heights were taken with a rigid measuring tape and the Body Mass Index (BMI) for each individual was calculated from the formula weight/height^2^ (kg/m^2^). The participants' BMIs were evaluated using the World Health Organization's classification system/guidelines/protocol 23.

### Beck Depression Inventory

The depression status of the participants was determined using the Beck Depression Inevtory, developed by Beck, et al. in 1961. The scale measures the intensity of physical, emotional, cognitive, and motivational symptoms in depression, with the characteristics of self-assessment 24. For instance, Turkish validity reliability was carried out by Teğin 25 and Hisli 26 in Turkey. Each item on the inventory is comprised of twenty-one categories and has 4 options. The scores obtained from the BDI vary between 0 and 63. Scores between 0 and 9 are considered in the “normal range”, while 10–16 are considered “light”, 17 to 29 “moderate depression”, and 30 to 63 points indicate “severe depression.”

### Caffeinated Beverages Consumption

In order to determine the amount in milliliters of caffeinated beverages consumed over the last 6 months by individuals, photos were shown from Photographed Food Catalogue - Measurements and Quantities^27^ and the quantities were estimated. The frequency of intake of these specified quantities was determined daily and multiplied by the consumption frequency coefficient (each day: 1.0; 5–6 times a week: 0.78; 3–4 times a week: 0.5; 1–2 times a week: 0.21; once every 15 days: 0.06; once a month: 0.03; none: 0) in order to calculate the daily consumption amounts. One cup was measured as 200 mL for black tea and instant coffee, or as 55 mL for Turkish coffee (very strong black coffee served with the fine grounds in it).

The average caffeine intake of individuals was calculated using the caffeinated beverage amounts consumed daily and the caffeine contents of these beverages. Since traditional preparation of tea and Turkish coffee differs from preparation techniques used in other countries, the caffeine content of black tea and Turkish coffee was determined using the study of Hanci, et al.^28^ According to this study, the caffeine content of black tea and Turkish coffee was 80mg/100mL and 80mg/55mL, respectively. The amount of caffeine in the remaining caffeine-containing beverages (cola, energy drinks, etc.) was determined using either the food label of each product or information from the USDA database^28^,^29^.

### Statistical Analysis

In the analyses, BDI scores of <10 were classified as being within the normal range, while scores of ≥10 were classified as depression. Tea consumption frequency was categorized as cups/day: <1 (reference), 1–2, 2–3, 3–4 and >4, while caffeine intake was categorized as mg/day: 0–300 (reference), 300–450, 450–600, 600–750 and >750. Variables that have been found in previous studies to potentially be associated with beverage consumption and depression were accounted for in the logistic regression models 30–32. Multivariate odds ratios (OR) and confidence intervals (CI) were obtained, adjusting for sex, age (10-year groups), BMI (kg/m2: <18.5, 18,5–25, ≥25), education level (Illiterate, elementary school, high school, and bachelor's degree), marital status (married and single), income level (low, middle, and high), smoking status (smoker or nonsmoker), alcohol consumption (yes or no), regular physical exercise (yes or no), diastolic and systolic blood pressures (normal, high-normal, and hypertension, according to the European Society of Cardiology's guidelines33). An alpha level of <0.05 was used as the threshold for statistical significance. All analyses were calculated electronically using STATA software.

## Results

The general characteristics of the research participants, according to their BD scores, are presented in Table 1. The average caffeine intake of the participants was 629.5±418.8 mg. Average caffeine intake was higher in participants aged 30 to 39, elementary school graduates, married people, smokers, middle income earners, and those with higher diastolic blood pressure (p<0.05). The prevalence of depression risk (≥10 BD score) among these participants was 30.1%. Higher depression risk is associated with age, BMI, marital status, income, smoking, alcohol consumption, regular physical activity, and systolic blood pressure (p<0.05).

**Table 1:** General characteristics of the participants according to their caffeine intake and BD scores

Parameter	Caffeine intake (mg)	p value	BD score <10 (n:343)	BD score ≥10 (n:148)	p value
Sex					
Male	676.5±486.1	0.036^a^	66.2	64.2	0,670
Female	604.8±377.2		33.8	35.8	
Age					
20–29 years	581.0±427.0	0.006^a^	40.2	54.0	0.035^a^
30–39 years	708.3±404.4		32.4	22.3	
40–49 years	685.2±459.4		16.6	14.9	
50+ years	526.0±297.8		10.8	8.8	
BMI					
<18.5	596.2±335.9		3.5	2.0	0.007^a^
18.5–25	639.2±439.7	0.847	47.5	62.8	
≥25	620.5±399.8		49.0	35.2	
Education Level (%)					
Illiterate	515.6±242.3		6.2	2.0	0,069
Elementary school	702.2±412.6	0.016^a^	26.5	22.3	
High School	670.6±470.2		26.5	24.3	
Bachelor's degree	576.2±397.6		40.8	51.4	
Marital status (%)					
Married	663.9±417.2	0.019^a^	60.9	46.6	0,003^a^
Single	584.5±417.5		39.1	53.4	
Income (%)					
Low	583.1±385.8		37.0	56.1	0,000^a^
Middle	701.3±462.9	0.006^a^	46.4	29.7	
High	567.3±355.8		16.6	14.2	
Smoking (%)					
Yes	735.2±485.1	0.000^a^	24,5	34,5	0,023^a^
No	590.5±384.3		75,5	65,5	
Alcohol consumption (%)					
Yes	695.6±625.5	0.149	6.4	12.2	0,030^a^
No	623.6±395.8		93,6	87,8	
Regular physical activity (%)					
Yes	629.1±437.4	0.495	26.0	18.9	0,093
No	429.6±413.4		74.0	81,1	
Beck Depression Inventory Scores			4.0±2.7	18.5±8.0	0,000^a^
Systolic blood pressure					
Normal (<129 mmHg)	618.7±403.0		68.5	73.0	0.026^a^
High Normal (130–139 mmHg)	608.1±422.4	0.074	24.2	14.9	
Hypertension (≥140 mmHg)	767.8±510.1		7.3	12.1	
Diastolic blood pressure					
Normal (<85 mmHg)	629.4±419.4		79.9	71.6	0.133
High Normal (85–89 mmHg)	547.8±386.2	0.030^a^	12.5	17.6	
Hypertension (≥90 mmHg)	763.0±439.7		7.6	10.8	

a p<0,005

The mean consumption of beverages and caffeine intake is shown in Table 2. As expected, black tea was consumed by all of the participants (n:491) and also had the highest consumption level (620.1±90.4mL). Turkish coffee (52.1%), instant coffee (46.0%), and cola (37.1%) were also regularly-consumed caffeinated beverages by the population studied. Black tea was the main source of caffeine (576.4±407.4 mg).

**Table 2:** The mean consumption of beverages and caffeine intake

		Consumption Amount (mL)	Caffeine Intake (mg)

Beverages	n (%)	mean ± Sd	mean ± Sd
Black Tea	491 (100.0)	620.1±90.4	576.4±407.4
Instant Coffee	226 (46.0)	189.2±181.3	61.1±65.0
Turkish Coffee	256 (52.1)	37.0±30.5	32.8±25.7
Green Tea	33 (6.7)	159.2±124.5	30.7±28.6
Cola	182 (37.1)	118.5±159.0	10.9±14.7
Energy Drinks	7 (1.4)	143.4±185.8	42.4±49.8

Table 3 indicates that, in all models, black tea consumption levels higher than 1 cup and up to 4 cups, were protective against depression risk. Moreover, caffeine intake levels of 450–600 mg also reduce the risk of depression than lower or higher doses.

**Table 3:** Odds ratios and 95% confidence intervals of BD scores according to different black tea consumption and caffeine intake levels

Black Tea Consumption	< 1 cup n:23^a^/20^b^		1–2 cup n:73^a^/28^b^		2–3 cup n:68^a^/25^b^		3–4 cup	n:78^a^/26^b^	> 4 cup n:101^a^/49^b^		

	OR	95% CI	OR	95% CI	OR	95% CI	OR	95% CI	OR	95% CI	p value

Model 1	ref	ref	0.44	0.21 – 0.93	0.41	0.19 – 0.88	0.39	0.18 – 0.82	0.55	0.28 – 1.11	0.041
Model 2	ref	ref	0.44	0.20 – 0.93	0.40	0.18 – 0.86	0.42	0.20 – 0.91	0.59	0.29 – 1.20	0.003
Model 3	ref	ref	0.44	0.20 – 0.95	0.42	0.19 – 0.93	0.45	0.21 – 0.98	0.59	0.28 – 1.21	0.000

Caffeine intake	< 300 mg n: 58^a^/38^b^		300–450mg n:64^a^/26^b^		450–600mg n:77^a^/20^b^		600–750mg n:48^a^/18^b^		>750mg n:96^a^/46^b^		

	OR	95% CI	OR	95% CI	OR	95% CI	OR	95% CI	OR	95% CI	p value

Model 1	ref	ref	0.68	0.36 – 1.26	0.40	0.21 – 0.77	0.61	0.31 – 1.21	0.74	0.43 – 1.28	0.003
Model 2	ref	ref	0.64	0.34 – 1.20	0.42	0.22 – 0.82	0.70	0.35 – 1.40	0.80	0.46 -1.40	0.003
Model 3	ref	ref	0.57	0.30 – 1.09	0.39	0.20 – 0.77	0.64	0.31 – 1.31	0.71	0.40 – 1.27	0.000
Model 4	ref	ref	0.56	0.29 – 1.07	0.40	0.20 – 0.78	0.66	0.32 – 1.38	0.81	0.44 – 1.49	0.000

Model 1: Sex, age, BMI; Model 2: Model 1 + education level, marital status, income; Model 3: Model 2 + smoking status, alcohol consumption, regular physical activity, diastolic and systolic blood pressure; Model 4: Model 3 + Caffeine intake other than black tea

a BDI score<10

b BDI score≥10

1 cup: 200 mL

## Discussion

This study aimed to investigate the relationship of caffeine intake and black tea consumption, which is the main source of caffeine among the population of Turkey, with depression. Multiple studies have reported on the relationship between caffeine and depression 34–37. Our study strongly suggests that the participants' daily caffeine intake through beverages was 629.5±418.8 mg (and 450–600 mg/d caffeine level) has a protective effect against depression, according to BD scores. In addition, the prevalence of depression was lower in participants who consumed between 1 to 4 cups of black tea per day, after adjustment for potential confounders.

Similar to these findings, previous studies have supported inverse relationships between caffeine and depression. Smith, et al. determined that the prevalence of clinical depression was 4.1% in individuals who consumed more than 260 mg of caffeine daily, whereas it was 33.7% among those who did not consume caffeine (p<0.05) 34. In another study, a J-shaped relationship was found between caffeine intake and post-partum depression^38^. In other recent meta-analysis, it was shown that 68 to 509 mg of caffeine intake significantly decreased the risk of depression 39. The relationship between caffeine and depression varies with the amount of daily caffeine intake 4,13,38,39. Consistent with these findings, the present study found that 450–600mg of caffeine intake caused a 61% reduction in the risk of depression compared to a 0–300 mg caffeine intake. (Table 3).

The high levels of tea consumed could have developed in the consumer a tolerance for caffeine40. In that case, consumption above a certain amount, when continuous, could decrease the positive effects of caffeine. Since the effect of caffeine on depression varies according to the amount of caffeine intake, accounting for the habitual consumption of caffeine-containing drinks is also important.

In this study, the caffeine intake of participants was higher than it was in much of the existing literature^4^,^9^,^10^,^34^,^41^ and black tea was the major source of caffeine. Next to water, black tea is the most widely consumed traditional beverage in Turkey 17. Euromonitor International reported that black tea consumption in Turkey was the highest per person in the world 42. In several studies conducted in Turkey, black tea was found to be the most preferred beverage for breakfast and with snacks 18 as well as the most consumed beverage overall^17^. The findings that supports this information were also revealed in this study. Consumption of black tea was 620.1±90.4 mL, which exceeds that of any other beverage (Table 2).

The protective effect of tea against depression has been demonstrated repeatedly 13,16,43,44. Hintikka, et al. found an inverse correlation between tea consumption in adults and their scores on the BDI 13. Moreover, it was stated that the individuals who consume tea were less depressed than those who do not (p<0.05). The same study did not find features of depression in individuals who drank more than five cups of tea daily^13^. A similar study determined that those with high tea consumption levels had lower levels of symptoms of depression^43^. Moreover, in a meta-analysis evaluating this subject, an inverse relationship was found between tea consumption and depression, and also that those who consumed more than three cups of tea daily had reduced risk of depression by 37.0% 44. Consistent with existing literature, in this study, it was found that consumption of 1 to 4 cups of black tea decreased the risk of depression by approximately 55.0% (Table 3).

The mechanism underlying the relationship between tea consumption and a risk of depression is still under investigation. Some studies have illustrated that the relationship between caffeine and depression results from caffeine's impact on the nervous system 44,45. Caffeine and caffeine's largest metabolite adenosine receptors increase the flexibility of hippocampal CA2 neurons in the brain, reducing the risk of depression in individuals who consume coffee 46,47. It is generally considered that the safe daily dosage of caffeine intake is less than 500 mg 48 however according to the Dietary Guidelines for Americans 2015–2020, caffeine intake up to 400 mg per day can be acceptable 41. In addition to caffeine, beverages such as coffee and tea contain antioxidants and phytochemicals, which might be factors in low levels of depression 15. A recent mechanism study showed that black tea L-theanine, epigallocatechin gallate, and theaflavins associated with anti-depressive effects through multiple pathways. These pathways include up-regulation of ERK/CREB/BDNF signaling, down-regulation of NF-kB signaling, and modulating dopaminergic activity and the gut-brain axis 49.

The current literature examining associations between caffeine intake and depression has been mainly carried out in Western countries with high levels of coffee consumption or in Eastern countries with levels of high green tea consumption. Uniquely, Turkey has the highest per capita consumption level of black tea. Furthermore, the country's traditional brewing style (15–30 min. brewing) increases the caffeine and polyphenol content of black tea, which results in higher intakes of caffeine and polyphenols 50. As expected, the results of this study indicated that black tea had the highest consumption level among all caffeine-containing beverages. The consumption of 1 to 4 cups of black tea (and 450–600 mg caffeine intake) was protective against depression. This protective effect may be the result of bioactive components as well as of caffeine itself. To better understand this protective effect, more studies of people who consume high amounts of black tea are needed.

## Limitations of Study

The sample of this research may be expanded to produce better results that more fully describe the population. In our study, all of the participants were habitual black tea consumers which caused us to select our reference group from the lowest level tea consumers. Adding a “non-consumer” group may improve the results of further studies. Together with the food consumption frequency of the participants, the evaluation of their food consumption records will also more accurately determine their daily caffeine intake. Throughout the research process, the participants were not examined, nor were sub-clinical depression diagnoses recorded by a physician. Personal statements regarding the symptoms of depression were taken as the basis. A more robust diagnostic method should be used in further studies.
